# Chromosome Genome Assembly of *Cromileptes altivelis* Reveals Loss of Genome Fragment in *Cromileptes* Compared with *Epinephelus* Species

**DOI:** 10.3390/genes12121873

**Published:** 2021-11-24

**Authors:** Yang Yang, Lina Wu, Zhuoying Weng, Xi Wu, Xi Wang, Junhong Xia, Zining Meng, Xiaochun Liu

**Affiliations:** 1 State Key Laboratory of Biocontrol, Life Sciences School, Sun Yat-sen University, Guangzhou 510275, China; yangy595@mail2.sysu.edu.cn (Y.Y.); wuln5@mail2.sysu.edu.cn (L.W.); wengzhy5@mail2.sysu.edu.cn (Z.W.); wuxi577@126.com (X.W.); wangx265@mail2.sysu.edu.cn (X.W.); xiajunh3@mail.sysu.edu.cn (J.X.); mengzn@mail.sysu.edu.cn (Z.M.); 2 Southern Laboratory of Ocean Science and Engineering, Zhuhai 519000, China

**Keywords:** humpback grouper, *Cromileptes altivelis*, genome, evolution, collinearity

## Abstract

The humpback grouper (*Cromileptes altivelis*), an Epinephelidae species, is patchily distributed in the reef habitats of Western Pacific water. This grouper possesses a remarkably different body shape and notably low growth rate compared with closely related grouper species. For promoting further research of the grouper, in the present study, a high-quality chromosome-level genome of humpback grouper was assembled using PacBio sequencing and high-throughput chromatin conformation capture (Hi-C) technology. The assembled genome was 1.013 Gb in size with 283 contigs, of which, a total of 143 contigs with 1.011 Gb in size were correctly anchored into 24 chromosomes. Moreover, a total of 26,037 protein-coding genes were predicted, of them, 25,243 (96.95%) genes could be functionally annotated. The high-quality chromosome-level genome assembly will provide pivotal genomic information for future research of the speciation, evolution and molecular-assisted breeding in humpback groupers. In addition, phylogenetic analysis based on shared single-copy orthologues of the grouper species showed that the humpback grouper is included in the *Epinephelus* genus and clustered with the giant grouper in one clade with a divergence time of 9.86 Myr. In addition, based on the results of collinearity analysis, a gap in chromosome 6 of the humpback grouper was detected; the missed genes were mainly associated with immunity, substance metabolism and the MAPK signal pathway. The loss of the parts of genes involved in these biological processes might affect the disease resistance, stress tolerance and growth traits in humpback groupers. The present research will provide new insight into the evolution and origin of the humpback grouper.

## 1. Introduction

The humpback grouper (*Cromileptes altivelis*), also called the mouse grouper, belongs to the *Cromileptes* genus of Epinephelidae. The genus only consists of the one species due to its special morphology compared to other groupers. Humpback groupers are mainly distributed in Western Pacific water [1]. The natural population of the humpback grouper is decreasing all over the world due to overfishing, climate change and habitat destruction (IUCN, https://www.iucnredlist.org/species/39774/100458943, accessed on 20 May 2021). Underwater surveys carried out in many areas, including Indonesia, Australia and New Caledonia, suggest that the humpback grouper is extremely rare and patchily distributed in reef habitats. The humpback grouper is one of the most expensive species in all of the grouper species in the live marine fish trade; the price has reached 130 USD per kilogram owing to its tender meat, beautiful color, special shape and rarity (IUCN). Though the artificial culture of the humpback grouper has been successful in recent years [2], the price is still high due to its low growth rate and low output of larva fish. Molecular-assisted selection based on the genome information is essential to increase the growth rate of humpback groupers in future breeding. 

The humpback grouper possesses special morphology compared with *Epinephelus* species. It has an extremely small anterior part of the head and a moderately deep body which forms a concave dorsal profile on the postorbital part, hence why it is also called humpback grouper. Moreover, it possesses a subuliform head and basiconic jaw without canine teeth so it is also widely called a mouse grouper. Generally, grouper species possess 7–11 dorsal spines. Just four groupers possess 10 dorsal spines, aside from the humpback grouper, including *Epinephelus snalogus*, *Hyporthodus exsul* and *H. nigritus*. Based on molecular phylogenetic analysis of mitochondrial and nuclear genes of groupers, the humpback grouper is separated from *Epinephelus* species [3,4,5,6]. However, the growth ratio of humpback groupers is far less than the most closely related *Epinephelus* species, such as *E. lanceolatus* and *E. fuscoguttatus*. The speciation and evolution of humpback groupers is a controversial issue. At present, no valid genome and transcriptome have been reported for the species. Lack of genetic resources seriously hinders the research about humpback groupers. Recently, several grouper species genomes, including red-spotted grouper (*E. akaara*) [7], giant grouper (*E. lanceolatus*) [8], leopard coral grouper (*Plectropomus leopardus*) [9], kelp grouper (*Epinephelus moara*) [10] and brown-marbled grouper (*Epinephelus fuscoguttatus*) [6], have been published. These high-quality genomes provide essential genetic resources for better understanding of the evolution and phylogeny of grouper species. In the present study, the assembly of the humpback grouper genome is important for further research on the speciation, evolution, molecular-assisted selection and the developmental mechanism of special shape.

## 2. Materials and Methods

### 2.1. Sample Collection, Library Construction and Sequencing

**Figure 1:**
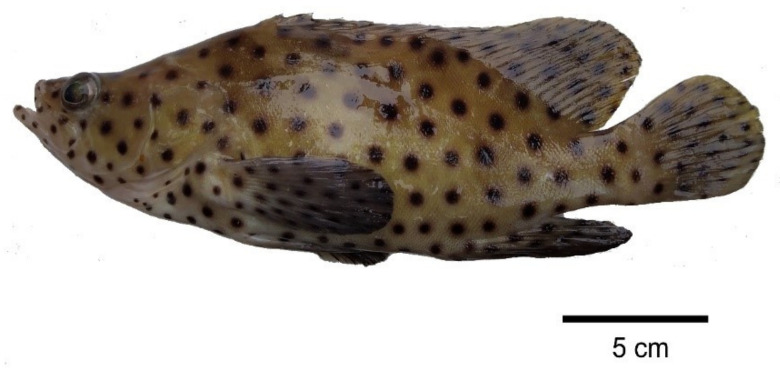
The characteristic of humpback grouper (*Cromileptes altivelis*).

A humpback grouper with body weight of 183.0 g and total length of 25.4 cm (Figure 1) was collected from Chenhai Aquatic Co., Ltd. (Hainan, China). The fish was immediately dissected after anesthesia with MS-222. White muscle tissue in the dorsal was sampled and immediately stored in liquid nitrogen, which was used for genomic DNA sequencing and Hi-C library construction. Moreover, ten tissues, including skin, muscle, liver, kidney, brain, intestine, fat, spleen, heart and gill, were collected and stored in RNAlater for transcriptome sequencing. 

Total DNA was extracted from white muscle tissue with a TIANamp Marine Animals DNA Kit (Tiangen Biotech Co., Ltd., Beijing, China). Quality and quantity of total DNA were determined by NanoDrop 2000 (Thermo Fisher Scientific Inc., Waltham, MA, USA). A paired-end sequencing library with insert length of 350 bp was constructed using a TruSeq Nano DNA LT Library Preparation Kit (Illumina, San Diego, CA, USA). The obtained library was then sequenced on Illumina HiSeq X Ten platform. 

Genome DNA was broken into fragments by Covaris and was recycled by AMpure PB beads (Pacific Biosciences, Menlo Park, CA, USA). A SMRTbell library was constructed using SMRTbell Template Prep Kit (Pacific Biosciences, USA), according to the manufacturer’s instruction and was sequenced on PacBio Bioscience Sequel platform (Pacific Biosciences, USA). 

Muscle samples were fixed with fresh paraformaldehyde and then DNA–protein bonds were created. The Mbo I restriction enzyme was used to digest the DNA and the overhanging 5’ ends of the DNA fragments were repaired with a biotinylated residue. The fragments, closed to each other in the nucleus during fixation, were ligated and the denatured proteins were removed. The Hi-C fragments were further sheared by sonication and then pulled down with streptavidin beads. The library was sequenced on an Illumina HiSeq X Ten platform with PE150 strategy.

Total RNA of the 10 tissues (approximately 80 mg of each) was extracted using RNAiso reagents (Takara, Dalian, China), following the manufacturer’s instructions. The quantity and quality of RNA samples were determined using a microplate spectrophotometer (BioTek Company, Winooski, VT, USA) and electrophoresis which was conducted using 1% agarose gel. Total RNA of the 10 tissues was mixed with equal amounts to generate a mixed RNA pool. An RNA-seq library was prepared using NEBNext UltraTM RNA Library Prep Kit (NEB, USA), following the manufacturer’s protocols. The library’s quality and quantity were measured using Agilent 2100 Bioanalyzer (Agilent Technologies, Santa Clara, CA, USA). Finally, the libraries were sequenced on Illumina-Hiseq 2000 platform with PE150 paired-end approach. A total of 119.33 Gb clean data was obtained with depth of 114.26× (Table 1).

### 2.2. Genome Assembly

We estimated the main genome characteristics of the humpback grouper through k-mer frequency distribution analysis [11]. After filtering, the clean data were used to estimate genome size and heterozygosity using 19-mer. 

For the PacBio sequencing data, after removing low-quality reads, clean data were corrected and assembled using Canu version 1.8 with parameters of genomeSize = 107,000,000, corOutCoverage = 80, corMhapSensitivity = low and correctedErrorRate = 0.025 [12]. Wtdbg was used to assemble genome by constructing fuzzy Brujin graph (https://github.com/ruanjue/wtdbg accessed on 20 May 2021). Quickmerge [13] was used to merge assemblies produced by Canu and wtdbg to produce a more contiguous assembly with a parameter of -hco 5.0 -c 1.5 -l 100,000 -ml 5000. In simple terms, contigs from Canu as query input and contigs from wtdbg as reference input are aligned through mummer version 4.0 [14]. The assembled genome was polished using Pilion version 1.22 [15]. For estimating genome completeness, Illumina data were mapped back to the humpback grouper genome to calculate the mapping rate using BWA v0.7.17 [16]. The genome integrality was verified based on the Core Eukaryotic Genes Mapping Approach (CEGMA) database [17] and Benchmarking Universal Single-Copy Orthologs (BUSCO) database [18]. 

### 2.3. Pseudochromosome Construction

The sequencing data of Hi-C were filtered to remove low-quality reads using Fastp version 0.12.6 [19]. The clean reads were aligned to draft genome of humpback grouper using bowtie2 version 2.3.2 [20] with end-to-end model and a parameter of very-sensitive. The draft genome was broken into 50 Kb fragments and reassembled with correct cluster, order and orient of the contigs using Lachesis with parameters of CLUSTER_MIN_RE_SITES = 100; CLUSTER_MAX_LINK_DENSITY = 2; CLUSTER_NONINFORMATIVE_RATIO = 2; ORDER_MIN_N_RES_IN_TRUN = 15; and ORDER_MIN_N_RES_IN_SHREDS = 15 [21]. 

### 2.4. Repeat Annotation

Repetitive regions of the wild humpback grouper genome were identified by de novo and homology predictions. Transposable elements (TEs) were identified using LTR Finder version 1.05 [22], RepeatScout v1.0.5 (http://www.repeatmasker.org accessed on 20 March 2020) and PILER v1.0 [23]. The TEs were classified and annotated using PASTEClassifier version 1.0 using TEdenovo pipeline [24]. Coding sequences were removed from the predicted repeat sequences through alignment to the SwissProt database using blastx with e-value < 1×10^−4^, identity > 30, coverage > 30%, and length > 90 bp. RepeatMasker v4.0.5 [25] was used to identify repeats based on the RepBase library version 19.06 [26] of known transposable elements (TEs). 

### 2.5. Genome Prediction and Annotation

Predicted non-coding RNAs includes micro RNA (miRNA), ribosomal RNA (rRNA) and transfer RNA (tRNA). tRNA was predicted using tRNAscan-SE v1.3.1 [27]. microRNA and rRNA were predicted using Blast+ version 2.2.31 (https://blast.ncbi.nlm.nih.gov/Blast.cgi?CMD=Web&PAGE_TYPE=BlastDocs&DOC_TYPE=Download, accessed on 20 March 2021) with e-value < 1 × 10^−5^ based on the Rfam database (ftp://ftp.ebi.ac.uk/pub/databases/Rfam accessed on 20 May 2021) [28]. miRNAs were predicted using Infenal version 1.1.1 [29] based on miRBase version 21 [30]. 

Protein-coding genes were predicted using three methods, i.e., de novo prediction, homologous sequences prediction and RNA-seq-assisted methods. For de novo prediction, the genome without repeat regions was applied to generate gene structures using Genscan [31], Augustus version 2.4 [32], GlimmerHMM version 3.0.4 [33], GeneID version 1.4 [34] and SNAP version 2006-07-28 [35]. Four fish species genomes and their annotation files, including tilapia (*Oreochromis niloticus*, GCF_001858045.2), zebra fish (*Danio rerio*, GCF_000002035.6), large yellow croaker (*Larimichthys crocea*, GCF_000972845.2) and Atlantic salmon (*Salmo salar*, GCF_000233375.1) were downloaded from Genbank database. The genome of humpback grouper was aligned to these genomes downloaded from NCBI using GeMoMa [36] to obtain the exact exons, introns and splice sites [36]. RNA-seq data from ten tissues were aligned to the genome using HISAT2 version 2.0.4 [37] and were assembled using Stringtie version 1.2.3 [38]. Open reading frames (ORFs) were predicted using PASA version 2.0.2 [39], TransDecoder version 2.0 (https://github.com/TransDecoder/TransDecoder, accessed on 20 March 2021) and GeneMarkS-T version 5.1 [40]. The genes predicted by the three methods were merged using EVM (http://evidencemodeler.sourceforge.net/, accessed on 20 March 2021) [41]. 

The putative functions of predicted genes were annotated by aligning them to NR [42], KOG [43], GO [44], KEGG [45] and TrEMBL [46] databases using Blast+ version 2.2.31. 

### 2.6. Evolution Analyses

For identifying gene family, eight fish species including seven grouper species (giant grouper (*E. lanceolatus*), orange-spotted grouper (*E. coioides*), brown-marbled grouper (*E. fuscoguttatus*), humpback grouper (*Cromileptes altivelis*), kelp grouper (*E. moara*), red-spotted grouper (*E. akaara*) and leopard coral grouper (*Plectropomus leopardus*)) and an outgroup large yellow croaker (*Larimichthys croceawere*) were collected for comparison using all-to-all BLAST with 1 × 10^−5^ of e-value. The orthogroups of eight species were predicted using Orthofinder version 2.3.7 [47]. 

The phylogenetic tree was constructed using shared single-copy genes among the eight fish species mentioned above. Protein sequences of these single-copy genes were aligned using MAFFT version 7.394 [48]. Gblocks version 0.91b [49] was used to remove low-quality alignments. The phylogenetic tree was constructed using RAxML software version 8.0.9 [50] and IQ-tree version 1.6.11 [51] with bootstrap value of 1000. 

The genomic collinearity analyses of brown-marbled grouper and other groupers were carried out using MCScanX [52]. The divergence time at each tree node was predicted using MCMCtree in PAML package version 4.9 [53]. The two calibration times including *E. akaara* vs. *E. fuscoguttatus* with 15.2~28.5 Myr and *L. crocea* vs. *P. leopardus* with 99~127 Myr were obtained from the TimeTree database [54]. 

## 3. Results

### 3.1. Sequencing and Genome Assembly

**Figure 2:**
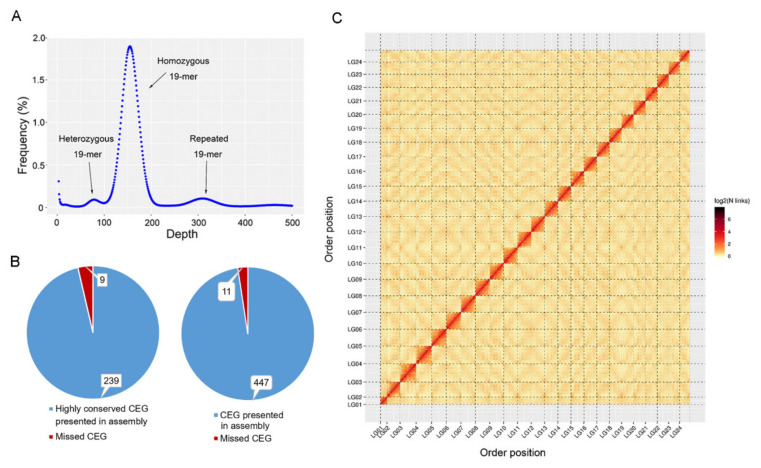
Genome assembly of humpback grouper. (**A**) Estimation of genome size, repeat content and heterozygous by survey using 19-mers in humpback grouper; (**B**) Verification of genome integrality based on the Core Eukaryotic Genes Mapping Approach (CEGMA) database; red parts and blue parts indicated missed and presented core eukaryotic genes (CEG) in humpback grouper genome, respectively; (**C**) The genome contig contact matrix; the blocks indicated the contacts between linkage groups; color depth indicated the degree of contacts.

Firstly, next-generation sequencing was applied to estimate the genome characteristics, including size, heterozygosity and repeat ratio, using the k-mer method. A total of 196.19 Gb clean data (184×) was obtained, with Q20 of 96.80% and Q30 of 91.90% (Table 1). The main peak of 19-mer in frequency distribution was at a depth of 152× (Figure 2A). The predicted genome size of the humpback grouper is 1.07 Gb with repeats of 29.53%. The heterozygosity and GC content were 0.09% and 41.2%, respectively. 

For genome assembly, a total of 119.33 Gb PacBio data (114.26×) and 100.88 Gb Hi-C data were obtained. The clean data included 6,672,321 reads with N50 of 27.96 Kb and an average length of 17.89 Kb (Table 1). A draft genome was assembled with 470 contigs with a length of 1.04 Gb and a contig N50 of 18.09 Mb using Canu and wtdbg (Table 1).

The genome quality was assessed by illumina data using BWA. The results showed that 98.29% of reads mapped into the draft genome, while 96.25% of reads properly mapped into the genome. The genome was aligned against the CEGMA database, which was constructed by a set of 458 core eukaryotic genes (CEGs) that are present in a wide range of taxa. A total of 447 (97.60%) CEGs were mapped (Figure 2B). The genome was then aligned to a reference gene set from the BUSCO database, which was constructed from 20 fish species, consisting of 4584 genes. A total of 4351 (94.92%) genes were mapped completely.

The Hi-C data was applied to correct misjoins, order and orientation in the draft genome. At last, a total of 283 contigs with 1,013,358,489 bp were obtained. Of them, 163 (57.60%) contigs were anchored into 24 pseudochromosomes with 1.012 Gb (99.82%) (Figure 2C). In the anchored contigs, 143 (87.73%) contigs with 1.011 Gb (99.91%) were correctly assembled into chromosomes (Table 1). 

### 3.2. Annotation

A total of 1,618,118 repeat sequences were predicted with 380.56 Mb, which account for 37.55% of the humpback grouper genome (Table S1). Of them, 2184 microsatellite sequences with 1,738,061 bp were detected, which account for 0.17% of the humpback grouper genome.

For non-coding RNAs, a total of 490 miRNA, 530 rRNA and 1239 tRNA were predicted.

For protein-coding genes, a total of 26,037 genes were predicted (Table S2); the total length and average length of these genes were 443.64 Mb and 17,038.97 bp. There were 252,243 exons, 226,206 introns and 245,667 coding sequences in the whole genome (Table S3). A total of 25,243 (96.95%) genes were aligned into at least one database. A total of 12,734 (48.91%), 15,086 (57.94%), 16,598 (63.75%), 24,908 (95.66%) and 25,218 (96.85%) genes were mapped into the GO, KEGG, KOG, TrEMBL and NR databases, respectively.

### 3.3. Evolution analyses

**Figure 3:**
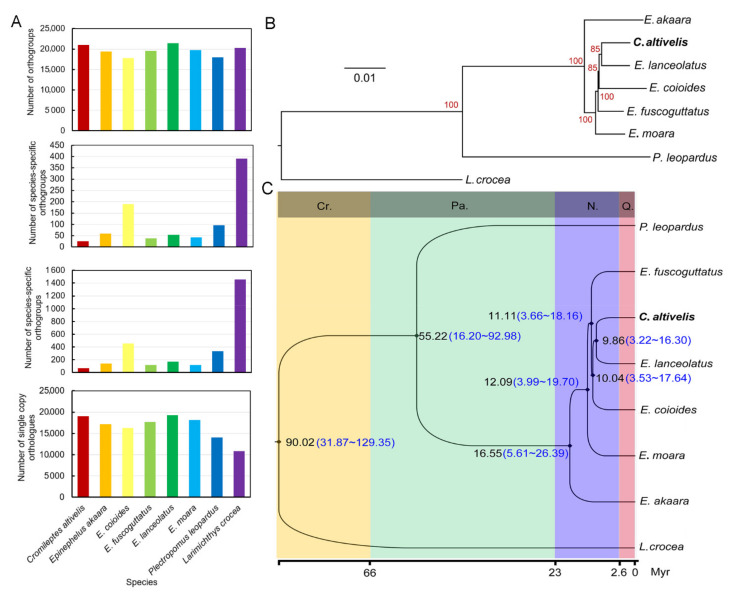
Analysis of divergence time of humpback grouper and other grouper species. Large yellow croaker (*L. crocea*) was set as outgroup. (**A**) The orthogroups statistics of the eight species; (**B**) phylogenetic tree of humpback grouper and other grouper species, red number indicated the bootstrap values; (**C**) Analysis of divergence time between humpback grouper and other grouper species using MCMCtree in PAML, the bold word indicated fish species used in the present study; two calibration times were obtained from TimeTree database; black number indicated the mean value of divergence time, blue number indicated the 95% confidence intervals of divergence times; Cr.—Cretaceous, Pa.—Paleogene, N. —Neogene.

The orthogroups of eight fish species were predicted using Orthofinder software. A total of 23,448 orthogroups were detected in all species. A total of 20,971 orthogroups were predicted in the humpback grouper. Of them, 25 species-specific orthogroups and 65 species-specific genes were detected (Figure 3A). Parts of these genes could be annotated and associated with myofiber structure and immunization. The number of shared single-copy orthogroups is 5066, which was applied in the establishment of the phylogenetic tree.

The phylogenetic tree from RAxML software was consistent with that from the IQ-tree software (Figure 3B and Figure S1): the humpback grouper and other *Epinephelus* species clustered into one branch with high bootstrap value (≥85). The giant grouper is the most closely related species to the humpback grouper with the shortest divergence time (3.22~16.30 Mya) compared to other grouper species. Then, the two groupers were clustered with the orange-spotted grouper, brown-marbled grouper, kelp grouper, red-spotted grouper and leopard coral grouper in turn (Figure 2B). The divergence time between the humpback grouper and all the *Epinephelus* species was less than 16.55 Mya, while the divergence time between *Epinephelus* species and the leopard coral grouper reached 16.20–92.98 Mya (Figure 3C). The results suggested that the humpback grouper may diverge from *Epinephelus* species. 

**Figure 4:**
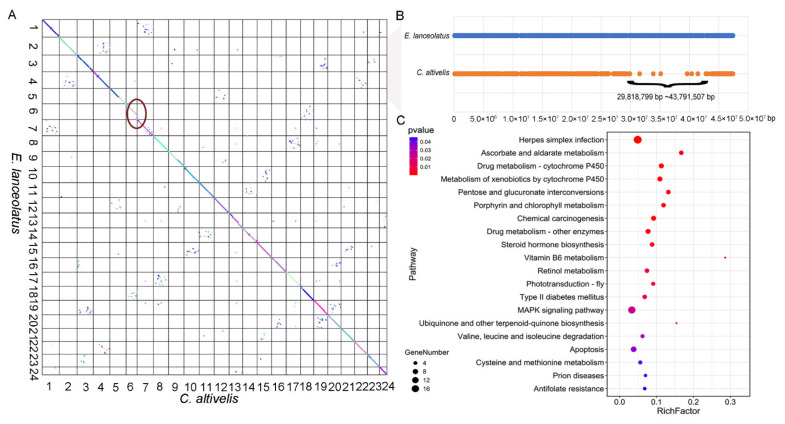
Collinearity analysis between humpback grouper and giant grouper. (**A**) Scatterplot of collinearity between humpback grouper and giant grouper, red circle showed the missing fragment in chromosome 6 of humpback grouper genome; (**B**) The distribution of genes in chromosome 6 of giant grouper and humpback grouper, multiple genes located in located in 29,818,799~43,791,507 bp of chromosome 6 were missed. (**C**) The KEGG enrichment analysis of missed genes.

The collinearity analyses were carried out using MCScanX. There was high collinearity between the humpback grouper and giant grouper (Figure 4A). However, there was a gap in chromosome 6 of the humpback grouper compared to the most closely related *Epinephelus* species (Figure 4A and Figure S2). Most of the genes located in 29,818,799~43,791,507 of the giant grouper genome were loosed in the humpback grouper (Figure 4B). Based on KEGG enrichment analysis (Figure 4C), the missed genes were mainly involved in immunity (IL1s, KRABs and JAK), substance metabolism (UGTs and GSTs) and MAPK signal transduction (CACNAs, FGF, EREG, MAPK1/3, MAPKAPK5 and MKP). 

## 4. Discussion

The humpback grouper, the only species in the *Cromileptes* genus of Epinephelidae, possesses special morphologies compared with other groupers. Thus, its speciation, evolution and phylogenetic position has attracted much attention. The high-quality chromosome-level genome of the humpback grouper provides an important foundation for the analyses of its origin and evolution.

In the research, a chromosome-level genome of the humpback grouper was assembled using PacBio sequencing and high-throughput chromatin conformation capture (Hi-C) technology. The heterozygosity of the genome was 0.09%. Its heterozygosity is lower than other grouper species, such as 0.375% in red-spotted grouper [7], 0.42% in leopard coral grouper [9] and 0.35% in brown-marbled grouper [6], which implies that the genetic diversity of the humpback is lower than other grouper species. The assembled genome was 1.013 Gb with 283 contigs, of which, a total of 143 contigs with 1.011 Gb in size were correctly anchored into 24 chromosomes. The genome size is similar with groupers in *Epinephelus*, such as 1.054 Gb in giant grouper [8], 1.047 Gb in brown-marbled grouper [6], 1.135 Gb in red-spotted grouper [7] and 1.08 Gb in kelp grouper [10]. 

The percentage of repeat contents in the humpback grouper was 37.55%, which was slightly higher than 34.6% of leopard coral grouper [9] and lower than 43.02% of red-spotted grouper [7], 41.1% of giant grouper [8] and 43.16% of brown-marbled grouper [6]. For protein-coding genes, a total of 26,037 protein-coding genes were predicted, of them, 25,243 (96.95%) genes could be functionally annotated. The results showed a high annotation rate. 

The specific genes were predicted using Orthofinder software. A total of 65 specific genes in the humpback grouper were obtained. These genes were mainly associated with myofiber structure and immunity, which may be involved in the growth and disease resistance of the humpback grouper. A total of 5066 shared single-copy orthogroups were obtained in the seven grouper species and the outgroup, which was higher than other research that used more outgroups [6,7,9]. The application of more single-copy orthogroups made the phylogenetic analysis more accurate. Based on the phylogenetic analyses of the humpback grouper and other *Epinephelus* species clustered into one branch with high bootstrap value, the giant grouper is the most closely related species of humpback grouper with a divergence time of 3.22~16.30 Mya. The divergence time between the humpback grouper and all the *Epinephelus* species was less than 16.55 Mya. Similar conclusions have been reported in several studies. For example, in the phylogenetic tree constructed by mitochondrial genome, the humpback grouper was clustered with the giant grouper as one clade, then clustered with all *Epinephelus* species as one clade [3]; in the phylogenetic tree based on cytochrome b gene, the humpback grouper was also clustered with the *Epinephelus* species as one clade [4]; and in the phylogenetic tree constructed by single-copy orthogroups, the humpback grouper was firstly clustered with the brown-marbled grouper, then was clustered with *Epinephelus* species [6].Though there were slight differences in topological structure, these results confirmed that *Cromileptes* originated from the *Epinephelus* genus.

There was high collinearity between the genomes of the humpback grouper and *Epinephelus* species. However, we found a gap in chromosome 6 which spanned 29,818,799~43,791,507 bp in the humpback grouper genome compared to the giant grouper. The result was also observed in the collinearity analysis between the brown-marbled grouper and humpback grouper [6]. Missed genes in the gap were mainly involved in immunity, substance metabolism and the MAPK signal pathway. Recently published research also reported that there were expansions of gene families involved in immunity in the brown-marbled grouper compared with the humpback grouper [6]. The change of genes involving immunity might induce a difference in disease resistance between the two species. For missed genes involved in immunity, interleukin 1s (IL1s) play important roles in innate inflammation through stimulating thymocyte proliferation and B-cell maturation and proliferation [55]. Tyrosine protein kinase (JAK) is involved in various processes such as metabolism, immunity and cell cycle control [56,57,58,59]. For substance metabolism, glucuronosyltransferases (UGTs) are essential factors in the elimination and detoxification of drugs and metabolism of endogenous and xenobiotics substances [60,61,62]; glutathione S transferases (GST) as detoxification enzymes could catalyze a combination of glutathione with electrophilic groups of substances, which play important roles in resistance to drugs, environmental pollutants and reactive oxygen species [63]. The MAPK signal pathway plays an important role in the regulation of cell proliferation [64]. The loss of the parts of genes involved in these biological processes might affect the disease resistance, stress tolerance and growth traits in the humpback grouper. In the missed fragment, the gene directly associated with morphology was not detected. The gene involved in the form of the humpback in the humpback grouper still need to be explored.

## 5. Conclusions

In the research, a high-quality chromosome-level genome of humpback grouper was assembled using PacBio sequencing and high-throughput chromatin conformation capture (Hi-C) technology, which will provide pivotal genomic information for future research of speciation, evolution and molecular-assisted breeding in humpback groupers. In addition, phylogenetic analysis, based on single-copy orthologues of grouper species, showed that the humpback grouper is included in the *Epinephelus* genus and clustered with the giant grouper to one clade with a divergence time of 9.86 Myr. Moreover, a gap in chromosome 6 of the humpback grouper was detected based on collinearity analysis; the missing genes were mainly associated with immunity, substance metabolism and the MAPK signal pathway. The loss of the parts of genes involved in these biological processes might affect the disease resistance, stress tolerance and growth traits in the humpback grouper.

## Figures and Tables

**Table 1:** The statistics of sequencing and assembled information of humpback grouper genome.

Raw Data	Reads Number	Reads Base (bp)	N50 (bp)	Max Length (bp)	GC Content (%)
Illumina data for annotation	39,031,897	11,658,953,034	150	150	49.7
Illumina data for survey	1,307,931,088	196,189,663,200	150	150	41.2
PacBio data	6,672,321	119,331,383,944	27,957	224,636	41.0
HiC data	336,775,366	100,882,400,830	150	150	42.6
Assembled data	Contig or Scaffold number	Genome size (bp)			
Survey	-	~1,070,000,000	-	-	41.2
Contig assembled using PacBio	470	1,044,397,337	18,092,086	49,150,803	41.3
Contig assembled using PacBio+Hi-C	283	1,013,358,489	18,269,829	49,150,803	41.2
Scaffold	164	1,013,370,389		52,436,080	41.2
Chromosome	24	1,010,598,072	43,466,351	52,436,080	41.2

## Data Availability

The chromosome-level genome assembly of the humpback grouper was deposited in the GenBank database with Whole Genome Shotgun projects: JAAWWW000000000. The raw data of NGS sequencing, Pacbio, Hi-C and RNA-seq for genome assembly of the humpback grouper were reserved in the GSA (Genome Sequence Archive) database and the accession numbers were CRA002771, CRA002772, CRA002773 and CRA002774.

## Supplementary Materials

The following are available online at https://www.mdpi.com/article/10.3390/genes12121873/s1, Figure S1: The phylogenetic tree of humpback grouper and other groupers using IQ-tree software, Figure S2: Collinearity analysis of *Cromileptes altivelis* and *Epinephelus coioides,* Table S1: The statistics of repeat information in humpback grouper genome, Table S2: Gene prediction of humpback grouper, Table S3: Construction statistics of predicted genes in humpback grouper genome.
