# Supplementary material for: Chromosome Genome Assembly of Cromileptes altivelis Reveals Loss of Genome Fragment in Cromileptes Compared with Epinephelus Species
Source: Genes (Basel). 2021 Nov 24;12(12):1873. doi: 10.3390/genes12121873 (PMC8701792; doi:10.3390/genes12121873)
Supplement: Supplementary file 1 [file genes-12-01873-s001.zip › genes-1463500-supplementary-done.pdf]

genes

Supplementary materials

Chromosome genome assembly of *Cromileptes altivelis* reveals *Cromileptes* separated from *Epinephelus*

Yang Yang[1], Lina Wu[1], Zhuoying Weng[1], Xi Wu[1], Xi Wang[1], Junhong Xia[1,2], Zining Meng[1,2*] and Xiaochun Liu[1,2*]

[1] State Key Laboratory of Biocontrol, Life Sciences School, Sun Yat-sen University, Guangzhou 510275, People's Republic of China
[2]Southern Laboratory of Ocean Science and Engineering, Zhuhai 519000, People's Republic of China
*Correspondence to Zining Meng, Email: mengzn@mail.sysu.edu.cn and Xiaochun Liu, Email: lsslxc@mail.sysu.edu.cn

Yang Yang

yangy595@mail2.sysu.edu.cn

Lina Wu

wuln5@mail2.sysu.edu.cn

Zhuoying Weng

wengzhy5@mail2.sysu.edu.cn

Xi Wu

Wuxi577@126.com

Xi Wang

wangx265@mail2.sysu.edu.cn

Junhong Xia

xiajunh3@mail.sysu.edu.cn

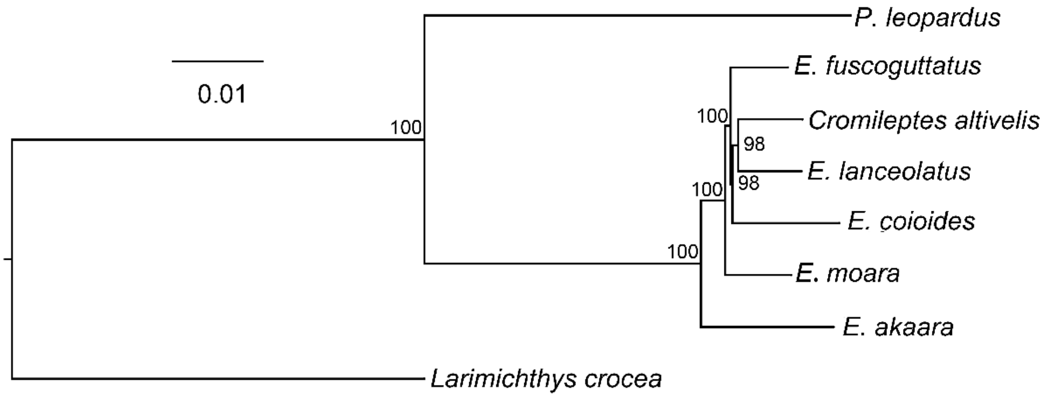

Figure S1 The phylogenetic tree of humpback grouper and other groupers using IQ-tree software.

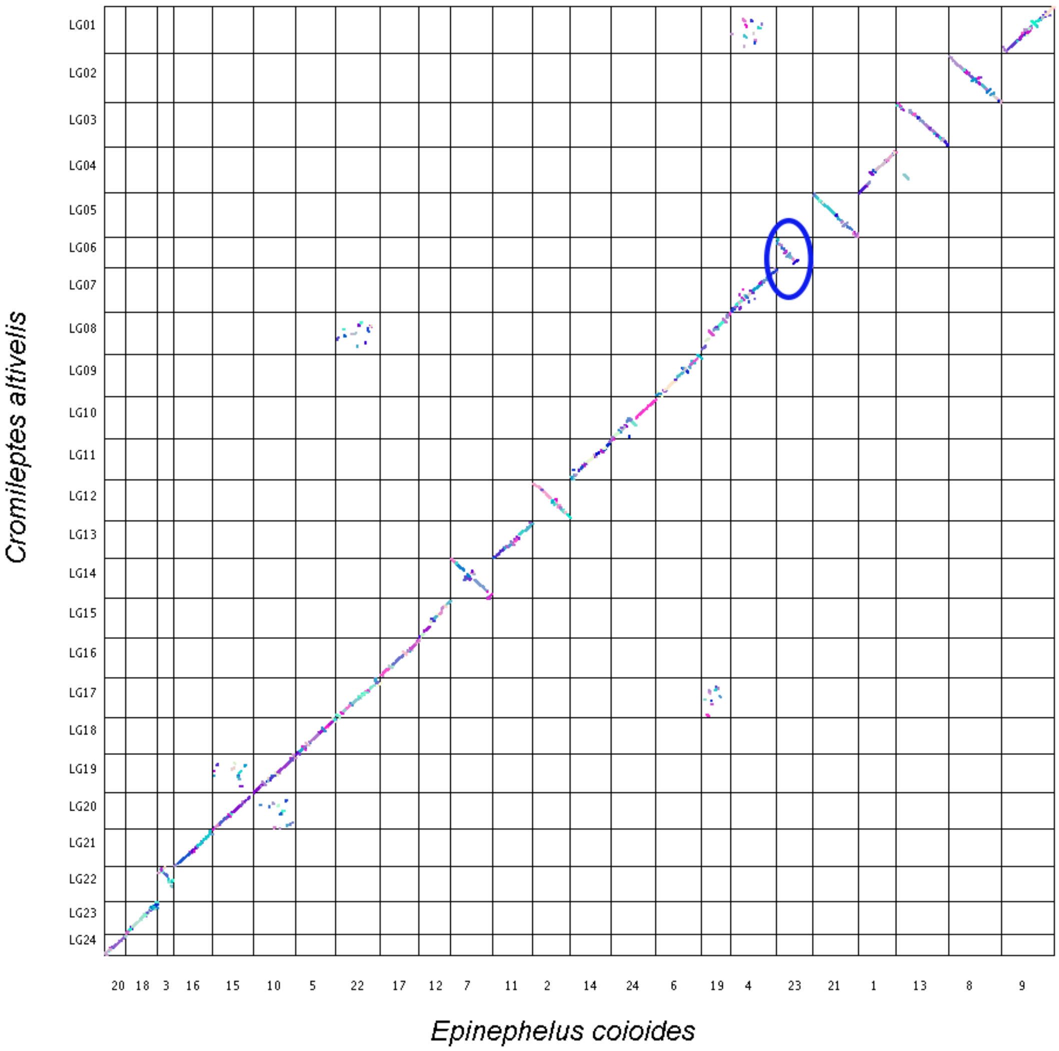

Figure S2 Collinearity analysis of *Cromileptes altivelis* and *Epinephelus coioides*

Table S1 The statistics of repeat information in humpback grouper genome

Classification	Type	Number	Length(bp)	Rate (%)
ClassI	Total	972,277	233,329,711	23.03
	DIRS	1,540	701,333	0.07
	LARD	624,310	134,784,136	13.3
	LINE	263,041	65,145,254	6.43
	LTR_Copia	4,726	607,374	0.06
	LTR_Gypsy	35,183	14,599,545	1.44
	LTR_Unknown	5,158	2,941,409	0.29
	PLE	30,264	9,453,329	0.93
	SINE	1,178	225,224	0.02
	TRIM	6,707	4,819,928	0.48
	Unknown	170	52,319	0.01
ClassII	Total	462,723	111,934,734	11.05
	Crypton	838	181,705	0.02
	Helitron	3,867	1,275,000	0.13
	MITE	10,701	3,107,721	0.31
	Maverick	1,925	867,351	0.09
	TIR	432,762	104,074,844	10.27
	Unknown	12,630	2,428,113	0.24
PotentialHostGene	-	16,610	3,314,773	0.33
SSR	-	2,184	1,738,061	0.17
Unknown	-	164,324	30,244,781	2.98
Total	-	1,618,118	380,561,917	37.55

Table S2 Gene prediction of humpback grouper

Method	Software	Species	Gene number
Ab initio	Genscan	-	31486
	Augustus	-	32786
	GlimmerHMM	-	97048
	GeneID	-	31103
	SNAP	-	42652
Homology-based	GeMoMa	*Danio rerio*	23619
		Salmo salar	26880
		Oreochromis niloticus	24970
		Larimichthys crocea	23491
RNAseq	TransDecoder	-	49859
	GeneMarkS-T	-	34675
	PASA	-	26883
Integration	EVM	-	26037

Table S3 construction statistics of predicted genes in humpback grouper genome

Type	Total number	Total length	Average length	Average number per gene
Gene	26,037	443,643,732	17,038.97	-
Exon	252,243	72,788,513	2,795.58	9.69
CDS	245,667	44,203,158	1,697.71	9.44
Intron	226,206	370,855,219	14,243.39	8.69
